# Seroprevalence of hepatitis B, C and D markers in indigenous patients seen at the Native American Outpatient Clinic of *Universidade Federal de São Paulo*

**DOI:** 10.31744/einstein_journal/2022AO6651

**Published:** 2022-04-07

**Authors:** Manuel Mindlin Lafer, Roberta Sitnik, Marcos Schaper dos Santos, Douglas Antônio Rodrigues, João Renato Rebello Pinho

**Affiliations:** 1 Hospital Israelita Albert Einstein São Paulo SP Brazil Hospital Israelita Albert Einstein, São Paulo, SP, Brazil.; 2 Universidade Federal de São Paulo São Paulo SP Brazil Universidade Federal de São Paulo, São Paulo, SP, Brazil.

**Keywords:** Hepatitis B, Hepatitis C, Hepatitis D, Serology, Antibodies, Indians, South American, Hepatitis B vaccines, Indigenous peoples

## Abstract

**Objective:**

To detect and treat cases of viral hepatitis B, C and D in patients seen at the Native American Outpatient Clinic of *Universidade Federal de São Paulo*.

**Methods:**

This sample comprised 81 indigenous recruited between 2018 and 2020. Volunteers were aged 7 months to 70 years (mean age of 28±20 years), belonged to 26 ethnic groups spanning the Brazilian territory and answered a questionnaire, which was attached to their medical records. Peripheral blood samples (20mL) were collected, transported to the Clinical Laboratory of *Hospital Israelita Albert Einstein*, processed, and tested for markers of viral hepatitis B, C and D.

**Results:**

In this study, 39 (48.1%) individuals were anti-HBs (+) only, 13 (16.0%) individuals were anti-HBs (+) and anti-HBc (+), and 28 (34.6%) individuals were negative for all markers. No anti-HBc IgM+ samples were found. No cases of hepatitis C and D were found.

**Conclusion:**

This analysis provided evidence of previous infection by the hepatitis B virus. These findings led to prescription of vaccination against hepatitis B to all participants who were negative for all viral hepatitis B markers, given records of prior hepatitis B vaccination were unreliable.

## INTRODUCTION

Brazilian indigenous peoples^(1)^ are extremely diverse. These people are distributed across 256 ethnic groups who speak approximately 150 languages. In 2010, a total of 896,917 Brazilian citizens described themselves as indigenous, accounting for 0.47% of the country’s total population. Of these, 324,834 were living in cities and 572,083 in rural areas. The 724 indigenous lands cover 12% of the Brazilian territory, although not as a continuum.

Indigenous persons can be found in all Brazilian regions, in widely different demographic situations.^(2)^ The North of the country comprises a sizeable portion of the indigenous population.^(3-5)^ In 1999, 34 *Distritos Sanitários Especiais Indígenas* (DSEIs) were created as part of the Public Health System (SUS - *Sistema Único de Saúde*), belonging to the Ministry of Health.

To offer Primary Health care in indigenous territories, DSEIs were connected to *Casas de Apoio à Saúde Indígena* (CASAIs). These are indigenous health centers created to support patients and companions outside the scope of basic services, generally in cities and locations with availability of high complexity medical services. Brazil is the only country to provide such a structure to its indigenous peoples.

**Figure 1:**
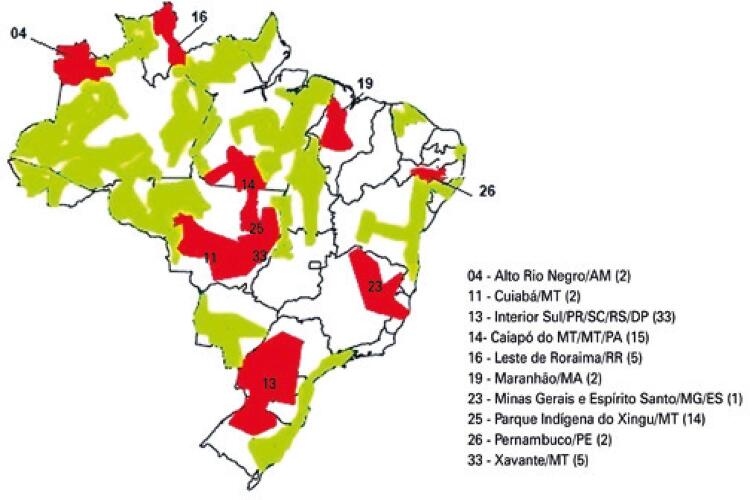
Brazilian Special Indigenous Health Districts included in the study

Source: adapted from Wikipédia, a enciclopédia livre. Distrito sanitário especial indígena. DESAI/FUNASA/MS, setembro 2003 [citado 2021 Jul 25]. Disponível em: https://pt.wikipedia.org/wiki/Distrito_sanit%C3%A1rio_especial_ind%C3%ADgena^(15)^

The São Paulo CASAI has beds for 20 patients and 20 companions and works in partnership with the Native American Outpatient Clinic of *Universidade Federal de São Paulo* (UNIFESP). In addition to delivering care to Brazilian indigenous persons throughout the country, São Paulo CASAI is the reference institution for DSEI *Litoral Sul*, which reaches all the way to the coast, to the east of Health District 13 (marked in green and red, respectively) (Figure 1).^(6,7)^*Universidade Federal de São Paulo* also delivers care and conducts research activities, community services, and training of human resources at Xingu Indigenous Park^(8,9)^and other locations.^(10)^

In 1989, hepatitis B vaccination became a routine public health activity in 13 cities in the Brazilian state of Amazonas.^(11)^ The prevalence of viral hepatitis B (HBV) and D in indigenous peoples living in the western Amazon rainforest is high, as demonstrated by studies carried out in Brazil and Peru, particularly with riverside populations,^(12)^ Colombia and Venezuela, where a highly lethal hepatitis D epidemic was reported in the Yucpa tribe, and Ecuador.^(11,13,14)^ The National Immunization Program (PNI - *Programa Nacional de Imunizações*) was established in 1973, and includes a specific schedule for Brazilian indigenous peoples.

Seroepidemiological surveys investigating viral hepatitis in Brazilian indigenous populations point to high rates of endemicity, morbity and mortality.^(11)^

The UNIFESP Native American Outpatient Clinic, officially founded in 1991, has been accumulating experience and expertise in care provision to Brazilian indigenous peoples since 1965. This study is based on projections from a database created in 2015, which comprises records of medical visits.

## OBJECTIVE

To detect and treat cases of viral hepatitis B, C and D in patients seen at the Native American Outpatient Clinic of *Universidade Federal de São Paulo.*

## METHODS

The present study is based on a digital database of UNIFESP’s Native American Outpatient Clinic. Data collected between January 2015 and October 2016 were used. During this time, 635 patients from 20 different ethnic groups were seen, of which 14 were represented by at least eight individuals.

The sample of 81 volunteer participants recruited sequentially by the principal investigator over the course of 1,749 multiprofessional visits (the same patient may have been seen more than once), taking place during the recruitment period and recorded in the above-mentioned database. After signature of an Informed Consent Form, secondary data of participants were collected via a questionnaire and analysis of physical and electronic medical records (as *per* the database: age in years, biological gender – male or female –, ethnicity, degree of kinship with the other participant in the case of family member – brother, son, spouse, relative etc. –, diagnosis that motivated the visit to the Native American Outpatient Clinic and respective International Classification of Diseases – ICD-10 – , and personal and family history.

Data were anonymized and uploaded into the REDCap Platform. Blood samples (20mL) were collected in dry tubes and tubes containing EDTA. The *Hospital Israelita Albert Einstein* (HIAE) Clinical Laboratory was in charge of weekly sample collection, transportation, storage and processing. The tests were carried out at the same laboratory, as *per* details given in table 1.

**Table 1:** Methods and kits used for hepatitis testing

Virus	Tests
Hepatitis B	HBsAg: kit HBsAg Qualitative II – Architect (Abbott), chemiluminescence
	AntiHBs: kit Anti-HBs – Architect (Abbott), chemiluminescence
	HBeAg: kit HBeAg – Architect (Abbott), chemiluminescence
	AntiHBe: kit Anti HBe – Architect (Abbott) chemiluminescence
	Anti-HBc: Total Kit Anti-HBc Total – Architect (Abbott), chemiluminescence
	Anti-HBc IgM: kit Anti-HBc IgM – Architect (Abbott), chemiluminescence
	HBV-DNA: viral load using the HBV CAP/CTM kit (Cobas AmpliPrep/Cobas TaqMan) v2.0, Roche Molecular Systems
Hepatitis C	Anti-HCV: Kit Anti-HCV – Architect (Abbott), chemiluminescence
	HCV-RNA: viral load using the HCV CAP/CTM kit (Cobas AmpliPrep/Cobas TaqMan) v2.0, Roche Molecular Systems
Hepatitis D	Anti-HDV: kit LIAISON XL MUREX Anti-HDV (Diasorin), chemiluminescence
	HDV-RNA: RealStar^®^ HDV RT-PCR Kit 1.0 Altona

### Patients

From October 4, 2018 to February 19, 2020, 81 volunteers were recruited at UNIFESP Native American Outpatient Clinic to participate in the research project “Complete Allelic Identification and HLA Gene and Viral Hepatitis Frequency in Brazilian Native American Populations*”.* Following analysis by the Ethics and Research Committees of partner organizations, and submission to the National Committee of Ethics and Research (Conep), which is responsible for studies involving Brazilian indigenous populations, this project was approved (#. 2.506.758 and CAAE: 73692717.1.000.5505 for UNIFESP; #. 2.542.476 and CAAE: 73692717.1.3001.0071 for HIAE). The following inclusion criteria were adopted: Brazilian indigenous patients or companions seen at UNIFESP Native American Outpatient Clinic. Participants with autoimmune disorders were excluded. This study was self-funded.

### Statistical analysis

The data were analyzed using software (Excel and Minitab).

## RESULTS

In this sample comprising 81 participants recruited during the experimental period, 40 (49%) subjects were recruited from UNIFESP Native American Outpatient Clinic and 41 (51%) from CASAI São Paulo indigenous health center. The sample comprised 40 female (49%), aged 7 months to 70 years (mean age of 28±20 years; median age of 30 years). Of these, 66 (81.5%) were patients and 15 (18.5%) were companions. The sample analyzed in this study and the population seen at the outpatient clinic (1,749 visits) over the course of the experimental period shared the same profile with regard to of age, sex and selected DSEI percentages.

Distribution and territories corresponding to the ten DSEIs included in this study (in red) and remaining DSEIs (24, in green) are shown in figure 1. *Distritos Sanitários Especiais Indígenas* that contributed participants are listed in numerical order according to respective numerical designation. Numbers in brackets indicate the number of participants contributed by each DSEI.

Table 2 describes the hepatitis B virus markers found in this population.

**Table 2:** Serological profile of past or present viral hepatitis B infection in patients seen at the Native American Outpatient Clinic of *Universidade Federal de São Paulo*

n	Presence of serological markers of HBV infection			Absence of serological markers of HBV infection	
	Anti-HBc (+)			Anti HBc (-)/HBsAg (-)	
	HBsAg (+)	Anti HBs (+)	HBsAg (-)/ anti-HBs (-)	Anti-HBs ≥10mUI/mL isolated	Anti-HBs ≤10mUI/mL
81	0 (0)	13 (16.0)	1 (1.2)	39 (48.1)	28 (34.6)

Results expressed as n (%).

HBV: viral hepatitis B.

Regarding hepatitis B, there were three groups identified in our study: susceptible individuals, requiring vaccination (34.6%), vaccinated individuals with anti-HBs levels ≥10IU/mL (48.1%), and individuals with prior contact with HBV (16.0%).

### Hepatitis C virus

No serological or molecular markers of viral hepatitis C were detected.

### Hepatitis D virus

Not detected among the cases with HBV markers.

## DISCUSSION

Evidence of previous infection by the hepatitis B virus was found in 17.2% of participants in this sample, albeit with no active viral replication. There were no cases of viral hepatitis C or D. In 34.6% of cases, test results for all HBV markers were negative. These surprisingly high numbers motivated the prescription of hepatitis B vaccination, given records of prior hepatitis B vaccination were either unavailable or unreliable. Another important factor was the difficulty involved in vaccinating populations in their villages, due to the geographical, logistical and cultural constraints, and the need to train and transport the medical teams, while maintaining the vaccines at appropriate temperature-controlled storage conditions.^(16)^

The environmental theory, which emphasizes factors such as forced migration, conflict, psychosocial refugee stress, overpopulation (clusters), hunger (and malnutrition), poor hygiene conditions, and lack of medications (when these were discovered and distributed among colonizers), may explain the vulnerability of American indigenous people to infectious diseases and epidemics, especially in the Amazon region. In a complementary and not mutually exclusive manner the high degree of homozygosity due to the founder effect can be added to this explanation.^(16)^ These populations have poor access to Primary Health care and vaccines. A review study analyzing 59 American indigenous societies in the Amazon region which were ravaged by 17 epidemics, between 1875 and 2008, identified measles, influenza and malaria as major diseases with fatal outcomes, with 6% contribution of hepatitis.^(16)^ European colonial expansion and the contact with indigenous Americans led to a catastrophic depopulation, driven by the introduction of infectious diseases in societies with limited exposure and immunity (“virgin territories”). This is still applicable to more than 50 populations of susceptible, isolated indigenous Americans.^(16,17)^ These indigenous populations are targeted of infectious and parasitic diseases. Viral epidemics, such as influenza and measles, decimated entire tribes, killing thousands of people in short periods of time, when there was no immunity.^(16)^

The prevalence of HBV increases from the south to the north of Brazil. The Amazon region has one of the highest rates of HBV carriers worldwide.^(18)^ The lifestyle of Brazilian indigenous people living in this region and limited access to vaccination put these societies at risk of HBV and HVD (hepatitis virus D) infection.^(19-22)^ Working in partnership with the Ministry of Health, the *Instituto Evandro Chagas* (IEC), located in the state of Pará (PA), Brazil, began to collect specimens from patients with fever and jaundice, in the city of Lábrea, in the Amazon region, in 1951. In the following decade, five child deaths were investigated. Seroepidemiological studies investigating HBV and HDV in American indigenous populations spanned the period from 1989 (Munduruku ethnic group, Itaituba, PA) to 2004 (Parakanã and Apyterewa ethnic group and Xingu villages, Altamira, PA, Brazil).^(23)^ Data on HBV in American indigenous villages point to a familial disease affecting young individuals (5 to 12 years of age) related to means and time of contact with other populations – Brazilian indigenous groups and others – and also to habits (piercing, scratching, alcoholism, travel, skin lesions, scarification, tattooing and oral preparation of foods). Horizontal interfamilial and sexual transmission is thought to be more important than vertical transmission.^(24-26)^ Viral hepatites B and D were transmitted to Brazilian indigenous peoples living in the Amazon region via reusable syringes and needles used in large scale vaccination against yellow fever in the 1940s and 1950s, and vaccines were made with human plasma, as well as reusable lancets used in finger pricking for malaria diagnosis.^(27)^

With regard to hepatitis B, three groups identified in this study: susceptible individuals requiring vaccination, immunized individuals with anti-HBs levels ≥10IU/mL and, individuals with prior contact with HBV.

Full vaccination schedule (three doses of vaccine against HBV) was prescribed for susceptible individuals. Participants were contacted directly during scheduled booked visits (Native American Outpatient Clinic participants) or letters addressed to the DSEI of origin (CASAI participants). Records were updated in *Hospital São Paulo* files, which is linked to the Native American Outpatient Clinic.

Vaccination in villages involves some challenges, such as storing vaccines in temperature-controlled conditions over great distances, and use of means of transportation subject to seasonal variations (river levels are their navigability are greatly impacted by rainfall). Other challenges include appropriate identification of individuals, incomplete or missing personal information (identity card, taxpayer identification number, vaccine record card, and medical records) and attendance issues, given that visits are schedule by leaders using different methods, such as radio or motorboats (canoes with 30 or 40 HP outboard engines). Different cultural habits between tribes interfere with healthcare actions: moving villages, spontaneous travel or not traveling without notice, tensions between ethnic groups living in the same reserve. Of notice, false negative results may have been included, since levels of protective anti-HBs antibodies begin to decline within 1 year of vaccination, a phenomenon related to humoral immunity. Participants in this study belonged to 26 different ethnic groups and formed 25 families, which brings together common cultural and environmental factors involved in the epidemiology of hepatitis B and respective vaccination strategies. Evidence suggests surveillance and revaccination are needed, given the susceptibility to HBV and the lack of reliable vaccination records.

This study was based on a population of patients seen at the Native American Outpatient Clinic of UNIFESP, which provides care to indigenous people in partnership with DSEIs CASAI São Paulo and *Litoral Sul*. Findings of this study are consistent with previous investigations with heterogeneous and scattered Brazilian indigenous populations, conducted in the last four decades, involving large numbers of peoples and large territories.^(12,28)^

## CONCLUSION

In conclusion, viral hepatites B, C and D are a major public health concern, since these diseases affect many people and may have severe complications, in particular, liver cancer and cirrhosis. Testing for serological markers of these diseases and constant surveillance strategies must be emphasized, for appropriate monitoring of hepatitis B virus vaccine coverage and early detection and treatment of cases. Also important, vaccination against viral hepatitis B also prevents hepatitis D.

## References

[1] 1. United Nations. Department of Economics and Social Affairs Indigenous Peoples. United Nations Declaration on the rights of indigenous peoples. New York: Unite Nations; 2007. p. 32 [cited 2021 Mar 20]. Available from: https://www.un.org/development/desa/indigenouspeoples/declaration-on-the-rights-of-indigenous-peoples.html

[2] 2. Dias C. Direitos dos povos indígenas e desenvolvimento na Amazônia. Reb Rev de Estudios Brasileños. 2019;6(11): 49-60 [I Número Especial – Bioma Amazonia].

[3] 3. Santos VS, Coimbra Jr CE. Cenários e tendências da saúde e da epidemiologia dos povos indígenas no Brasil. In: Coimbra JR CE, Santos RV, Escobar AL (Org). Epidemiologia e saúde dos povos indígenas no Brasil. Rio de Janeiro: Fiocruz; 2003. p. 13-47.

[4] 4. Instituto Brasileiro de Geografia e Estatística (IBGE). Indígenas. Rio de Janeiro: IBGE; 2021 [citado 2021 Fev 8]. Disponível em: https://indigenas.ibge.gov.br

[5] 5. Instituto Socioambiental (ISA). Povos Indígenas do Brasil. Altamira (PA): ISA; 2021 [citado 2021 Fev 8]. Disponível em: https://www.socioambiental.org/pt-br/o-isa/programas/povos-indigenas-no-brasil

[6] 6. Pereira ER, Biruel EP, Oliveira LS, Rodrigues DA. A experiência de um serviço de saúde especializado no atendimento a pacientes indígenas. Saude Soc. 2014;23(3):1077-90.

[7] 7. Brasil. Ministério da Saúde. Distrito Sanitário Especial Indígena (DSEI). Brasília (DF): Ministério da Saúde; 2021 [citado 2021 Fev 8]. Disponível em: https://saudeindigena1.websiteseguro.com/coronavirus/dsei

[8] 8. Lafer MM, de Moraes-Pinto MI, Weckx LY. Prevalence of antibodies against hepatitis A virus among the Kuikuro and Kaiabi Indians of Xingu National Park, Brazil. Rev Inst Med Trop Sao Paulo. 2007;49(3):155-7. 10.1590/s0036-46652007000300004 17625692

[9] 9. Lafer MM, de Moraes-Pinto MI, Weckx LY. Prevalence of IgG varicella zoster virus antibodies in the Kuikuro and Kaiabi indigenous communities in Xingu National Park, Brazil, before varicella vaccination. Rev Inst Med Trop Sao Paulo. 2005;47(3):139-42. 10.1590/s0036-46652005000300004 16021286

[10] 10. Baruzzi RG. A universidade na atenção à saúde dos povos indígenas: a experiência do Projeto Xingu da Universidade Federal de São Paulo/Escola Paulista de Medicina. Saúde Soc. 2007;16(2):182-6.

[11] 11. Figueiredo JO. Hepatite B e D na área do Distrito Sanitário Especial Indígena do Alto Rio Solimões – aspectos associados à prevalência e ao programa de atendimento aos portadores [dissertação]. Manaus: Universidade do Estado do Amazonas; 2016 [citado 2021 Fev 8]. Disponível em: https://pos.uea.edu.br/data/area/dissertacao/download/25-7.pdf

[12] 12. Cabezas C, Braga W. Hepatits B virus and delta infection: special considerations in the indigenous and isolated riveside populations in the Amazon Region. Clin Liv Dis (Hoboken). 2020;16(3):117-22. Review. 10.1002/cld.1009 PMC7508778 33005393

[13] 13. Centers for Diseases Control and Prevention (CDC). Viral hepatitis. Atlanta (USA): CDC;2021 [cited 2021 Fev 8]. Available from: https://www.cdc.gov/hepatitis/

[14] 14. Hadler SC, De Monzo M, Ponzetto A, Anzola E, Rivero D, Mondolfi A, et al. Delta virus infection and severe hepatitis. An epidemic in the Yucpa Indians of Venezuela. Ann Intern Med. 1984;100(3):339-44. 10.7326/0003-4819-100-3-339 6696355

[15] 15. Wikipédia, a enciclopédia livre. Distrito sanitário especial indígena. DESAI/FUNASA/MS, setembro 2003 [citado 2021 Jul 25]. Disponível em: https://pt.wikipedia.org/wiki/Distrito_sanit%C3%A1rio_especial_ind%C3%ADgena

[16] 16. Walker RS, Sattenspiel L, Hill KR. Mortality from contact-related epidemics among indigenous populations in Greater Amazonia. Sci Rep. 2015;5:14032. 10.1038/srep14032 PMC4564847 26354026

[17] 17. Black FL, Hierholzer WJ, Pinheiro F, Evans AS, Woodall JP, Opton EM, et al. Evidence for persistence of infectious agents in isolated human populations. Am J Epidemiol. 1974;100(3):230-50. 10.1093/oxfordjournals.aje.a112032 4370011

[18] 18. de Paula VS, Arruda ME, Vitral CL, Gaspar AM. Seroprevalence of viral hepatites in riverine communities from the western region of the Brazilian Amazon Basin. Mem Inst Oswaldo Cruz. 2001;96(8):1123-8. 10.1590/s0074-02762001000800016 11784933

[19] 19. Bensabath G, Soares MC. A evolução do conhecimento sobre as hepatites virais na região amazônica: da epidemiologia e etiologia à prevenção. Rev Soc Bras Med Trop. 2004;37(Suppl 2):14-26. 10.1590/s0037-86822004000700003 15586892

[20] 20. Braga WS, Brasil LM, Souza RA, Castilho MC, Fonseca JC. Ocorrência da infecção pelo vírus da hepatite B (VHB) e delta (VHD) em sete grupos indígenas do Estado do Amazonas. Rev Soc Bras Med Trop. 2001;34(4):349-55. 10.1590/s0037-86822001000400007 11562728

[21] 21. Soares MC, Bensabath G. Tribos indígenas da Amazônia Oriental como população de risco para hepatite D (delta). Rev Inst Med Trop São Paulo. 1991;33(3):241-2. 1844542

[22] 22. Ramírez JD, Sordillo EM, Gotuzzo E, Zavaleta C, Caplivski D, Navarro JC, et al. SARS-CoV-2 in the Amazon region: a harbinger of doom for Amerindians. PLoS Negl Trop Dis. 2020;14(10):e0008686. Erratum in: PLoS Negl Trop Dis. 2021;15(2):e0009118. 10.1371/journal.pntd.0008686 PMC7595282 33119616

[23] 23. Nunes HM, Soares CP. Saúde indígena: contribuição da Seção de Hepatologia do Instituto Evandro Chagas desde a década de 1980. Rev Pan-Amaz Saude. 2016;7(Esp):71-82.

[24] 24. Coimbra Júnior CE, Santos RV, Yoshida CF, Baptista ML, Flowers NM, do Valle AC. Hepatitis B epidemiology and cultural practices in Amerindian populations of Amazonia the Tupi-Mondé and the Xavante from Brazil. Soc Sci Med. 1996;42(12):1735-43. 10.1016/0277-9536(95)00295-2 8783434

[25] 25. Azevedo RA, Silva AE, Ferraz ML, Marcopito LF, Baruzzi RG. Prevalência dos marcadores sorológicos dos vírus da hepatite B e D em crianças das tribos Caiabi e Txucarramãe do Parque Indígena do Xingu, Brasil Central. Rev Soc Bras Med Trop. 1996;29(5):431-9. 10.1590/s0037-86821996000500005 8966307

[26] 26. Grajcer B. Prevalência da infecção pelo vírus da hepatite B e resposta imune à vacina recombinante contra hepatite B com esquema adaptado em população menor de 15 anos do Alto Xingu [tese]. São Paulo: Universidade Federal de São Paulo; 2001. 105 f.

[27] 27. Fonseca JC. Histórico das hepatites virais. Rev Soc Bras Med Trop. 2010; 43(3):322-30. 10.1590/s0037-86822010000300022 20563505

[28] 28. Villar LM, Milagres FA, Lampe E, Cruz HM, Scalioni LP, Magalhães MA, et al. Determination of hepatitis B, C and D prevalence among urban and Amerindian populations from the Eastern Brazilian Amazon: a cross sectional study. BMC Infect Dis. 2018;18(1):411. 10.1186/s12879-018-3279-2 PMC6102873 30126364

